# Modifiable cardiovascular risk factors in Rheumatoid Arthritis

**DOI:** 10.12669/pjms.334.12798

**Published:** 2017

**Authors:** Abrar Ahmed Wagan, Syed Nayab Haider, Rukhsar Ahmed, Fuad Shafiq, Sadia Nasir

**Affiliations:** 1 Dr. Abrar Ahmed Wagan, FCPS. Department of Medicine, Central Park Medical College, Lahore, Pakistan; 2 Dr. Syed Nayab Haider, MRCP, FRCP. Department of Medicine, Central Park Medical College, Lahore, Pakistan; 3 Dr. Rukhsar Ahmad, FCPS. Department of Medicine, Central Park Medical College, Lahore, Pakistan; 4 Dr. Fuad Shafiq, MRCP, FRCP. Department of Medicine, Central Park Medical College, Lahore, Pakistan; 5 Dr. Sadia Nasir, MBBS. Department of Medicine, Central Park Medical College, Lahore, Pakistan

**Keywords:** Rheumatoid Arthritis, CVD, Cholestrol, HDL, TG, FRS

## Abstract

**Objective:**

To determine the frequency of modifiable cardiovascular risk factors in Rheumatoid Arthritis patients at tertiary care hospital.

**Methods:**

During this study 246 patients of Rheumatoid Arthritis were enrolled from outpatients department of Medicine of Central Park Medical College Hospital, Lahore from July 1, 2016 to January 31, 2017. Demographic data and questions related to study were noted. After 14 hours of fasting 5ml of venous blood was drawn for Cholesterol, triglycerides, HDL and blood sugar level. Blood tests were performed on COBAS c III (ROCHE), Framingham 10 year Risk score was calculated for every individual.

**Results:**

The mean age of male population was (50.2 ±7.5) and females (48.4±7.6) and female gender was common. Seventy eight (78%) of study population has one modifiable risk factor. Most frequent risk factor found in this study was BMI>30 in 48.4% (n=119), High LDL 43.5% (n=107), moderate to high FRS score 40.2% (n=99), Hypertension 37.4% (n=92), Diabetes Mellitus was present in 22.8% (n=56), while smoking was least frequent risk factors with frequency of 15.9% (n=39). Framingham cardiovascular risk score was significantly different, males were having higher mean 10 year risk score (19.7%) and females (8.7%) with (p-<0.01). Regression analysis revealed that older patients of Rheumatoid Arthritis with disease duration of more than seven years are four times more likely to have High Framingham risk score, moderate to high LDL and diabetes mellitus with significant high Odds ratio (p-value <0.05).

**Conclusion:**

Rheumatoid Arthritis patients are having increased chances of developing cardiovascular risk factors leading to cardiovascular events with male sex, increasing age and disease duration.

## INTRODUCTION

Rheumatoid arthritis is the most common inflammatory arthritis affecting 0.5 to 1% of general population worldwide.^1^ Several studies have documented increased morbidity and mortality in RA, in a Canadian study it was found that RA patients had a median survival 17 years shorter than expected for general population.^2^

RA is a high-grade inflammatory disease with an overlapping cellular and cytokine profile; thus, it has been hypothesized that rheumatoid inflammation is a major cause of the accelerated atherosclerosis observed in this patient population, there is increasing evidence that traditional non modifiable and modifiable cardiovascular risk factors also play an important role in the higher risk of coronary atherosclerosis and coronary heart disease (CHD) events (fatal myocardial infarction, CHD deaths) in patients with RA.^3^

Atherosclerosis (AT) was once considered to be a degenerative disease that was an inevitable consequence of aging. However, research in the last three decades has shown that AT is not degenerative or inevitable. It is an autoimmune-inflammatory disease associated with infectious and inflammatory factors characterized by lipoprotein metabolism alteration that leads to immune system activation with the consequent proliferation of smooth muscle cells, narrowing arteries, and atheroma formation. Both humoral and cellular immune mechanisms have been proposed to participate in the onset and progression of AT.^4^

Systemic inflammatory response in RA is critical to accelerated atherogenesis operating via accentuation of established and novel risk pathways and long term suppression of systemic inflammatory response should be effective in reducing the risk of coronary heart disease.^5^

The rationale of this study was most of Rheumatoid Arthritis are treated by nontraditional ways due to lack of awareness about the disease its course and its aggressiveness and extra articular manifestations which pose a serious threat to health of a patient. This scarcity of knowledge about the disease leads to injudicious use of corticosteroids and non-steroidal anti-inflammatory medicines results into risk of having complications like cardiovascular problems, which sometime leads to early and unexpected deaths.

## METHODS

This cross sectional study was conducted in outpatient department of Rheumatology division of Medicine Department at Central Park Medical College Hospital Lahore from July 1, 2016 to January 31, 2017 after the approval of Institutional review board. Written and informed consent were taken from each participant. RA was diagnosed according to 2010 American College of Rheumatology criteria.

Sample size was estimated using openepi sample size calculator by inserting the 80% prevalence of at least one modifiable risk factor in RA patients 3, with 5% margin of error and 95% confidence interval n = 246 patient for this study.

All those patients of rheumatoid arthritis who were seropositive for either Rheumatoid factor or Anti citrillinuted peptides or both were included in this study, their demographic data and disease duration number of Disease Modifying anti rheumatoid drugs usage asked in detail.

All those patients who are sero-negative Rheumatoid Arhtirtis, psoriatic arthritis, Systemic Lupus erythematous, scleroderma, osteoarthritis, or any other connective tissue disease, using lipid lowering drugs, or on biological Disease modifying drugs, previous history of Myocardial Infarction, stroke, transient ischemic attack, or coronary revascularization were excluded from the study.

Body Mass Index (weight measured using a beam balance scale and height measured by a stadiometer) was calculated by dividing weight (in kilogram) by square of height (in meters), all those who has BMI score of 30 or more than thirty were labeled as Obese.

Hypertension was assessed by taking Blood pressure(mercury sphygmomanometer) in sitting position after five minutes rest, three times and mean of two last readings was noted and if readings were of >140mmHg systolic or 90mmHg diastolic pressure was taken as hypertension either self-reported diagnosis or any patient who was taking anti-hypertensive. Smoking status was evaluated in detail about the number of years since smoking, number of cigarettes per day and active or Ex-smoker.

Diabetes Mellitus was labeled by measuring Fasting blood glucose level and one who has Fasting blood glucose level of >126 mg/dl was labeled as Diabetes mellitus and patients either self-reported diagnosis or using insulin and/or oral hypoglycemic drugs.

Serum Cholesterol, HDL (high density lipoprotein), TG(Triglycerides), were measured for every study participant; 5ml of blood sample was drawn by a trained phlebotomist after 14 hours of fasting than blood samples were analyzed on ROCHEIII laboratory. LDL(Low density lipoproteins) was calculated by this Friedewald formula, LDL = TC - HDL - TG/5.0 (mg/dL).

Framingham risk score was calculated for each individual through an online calculator by setting the parameters and categorized into Low (<10%), Moderate (11-19%), High risk category (>20).

Study participants were asked about the use of Homeopathic Medications currently/past for more than six months, positive history of more than six months use was taken as study parameter.

### Data Analysis

Data was stored and analyzed using SPSS version 16.0, mean and standard deviation are given for age, BMI, SBP, DBP, Cholesterol, TG, LDL, HDL, FRS, and disease scores for male and female RA patients. Independent sample t-test was used to compare the mean of these parameters between two groups.

Count and percentages are given for Hypertension, Hypertension with Medication, Diabetes Mellitus, Smoking, DMARDS and Homeopathic Medication with chi square association with gender group. Pie chart was used to display the count of modifiable cardiovascular risk factors in RA patients.

Logistic regression analysis was performed to give the association of Modifiable cardiovascular risk factors and other parameters with duration of RA disease, odds ratio and 95% confidence interval for odds ration also given in the table. All p-values less than 0.05 were considered as significant.

## RESULTS

In this study there were 246 participants, males 58 (23.6%) and females 188 (76.4%), with mean age of male group was (50.2 ± 7.5) and females (48.4 ± 7.6) years (p-value 0.045). There was significant difference between the duration of disease in males (10.2) and female (8.40) years (p-value 0.03). Body Mass Index (BMI) males (29.3%) and females (29.6%) with (p-value 0.71).

The mean systolic blood pressure was statistically different males (131.4 ± 14.8mmHg) females (126.2 ± 3.8mmHg) with (p-0.017), and mean diastolic blood pressures was significantly different males (85.9 ± 9.8mmHg) and females (82.7 ± 9.5mmHg) with (p-0.02). Serum cholesterol levels were significantly different between males (207.7 ± 44.3) and females (182.4 ± 33.3) with (p-<0.01). Low density lipoproteins (LDL) calculated by Freidwald formula was between males (118.2 ± 44.4) and females (95.3 ± 33.4) with (p-0.01). Framingham 10 year cardiovascular risk score was, males (19.7%) and in females (8.7%) with (p-<0.01).(Table-I)

**Table-I:** Basic Characteristic of Sample Data (n = 246).

*Characteristics*	*Gender*				*p-value*

	*Male (n=58)*		*Female (n=188)*		

	*Mean*	*S.D*	*Mean*	*S.D*	
AGE	50.62	7.50	48.45	7.06	0.045*
BMI	29.33	4.00	29.06	5.29	0.71
SBP	131.34	14.89	126.26	13.83	0.017*
DBP	85.90	9.84	82.71	9.05	0.02*
Cholestrol	207.07	44.39	182.40	33.27	<0.01*
TG	220.67	106.46	201.69	81.07	0.151
LDL (F)	118.27	44.47	95.31	33.47	<0.01*
HDL	45.05	6.99	45.33	6.84	0.78
Framingham Score	19.78	9.70	8.79	8.00	<0.01*
Disease Duration	10.21	5.98	8.40	5.45	0.03*

* p<0.05 was considered significant using independent sample t-test.

The prevalence of obesity it was found that males 53.4% (n=31) and 46% (n=88) were having BMI> 30 with (p-0.132). Hypertension was present in 50% (n=29) males 33.5% (n=63) females. Diabetes was present in males 24.1% (n=14) females 22.3% (n=42) with (p-0.08). Disease Modifying anti rheumatoid drugs in study participants methotrexate combination was more in use males 37.9% (n=22) and 60.1% (n=113) with (p-0.01). Use of homeopathic medication was males 75.9% (n=44) in females 57.4% (n=108) with (p-value 0.012). (Table-II)

**Table-II:** Comparative analysis of study parameters.

*Characteristics*		*Sex*				*p-value*

		*Male (n=58)*		*Female (n=188)*		

		*n*	*%*	*n*	*%*	
BMI levels	18 - 24.9 “Normal“	7	12.1	46	24.5	0.132
	25 - 29.9 “Overweight”	20	34.5	54	28.7	
	>30 “Obese”	31	53.4	88	46.8	
Hypertension	Yes	29	50.0	63	33.5	0.023*
	No	29	50.0	125	66.5	
Hypertension with medication	Yes	23	39.7	53	28.2	0.09
	No	35	60.3	135	71.8	
Diabetes Mellitus	Yes	14	24.1	42	22.3	0.08
	No	44	75.9	146	77.7	
Smoking	Yes	32	55.2	7	3.7	<0.01*
	No	26	44.8	181	96.3	
FRS	<10 “Normal”	15	25.9	132	70.2	<0.01*
	11-20 “Moderate”	12	20.7	36	19.1	
	>20 “High”	31	53.4	20	10.6	
DMARDS	MTX	7	12.1	17	9.0	0.01*
	SSZ,HQ	9	15.5	29	15.4	
	MTX Combination	22	37.9	113	60.1	
	Non MTX	15	25.9	24	12.8	
	No DMARDS	5	8.6	5	2.7	
Hakeem/homeo Medication	Yes	44	75.9	108	57.4	0.012*
	No	14	24.1	80	42.6	

* p<0.05 was considered significant using chi square test.

**Fig. 1:**
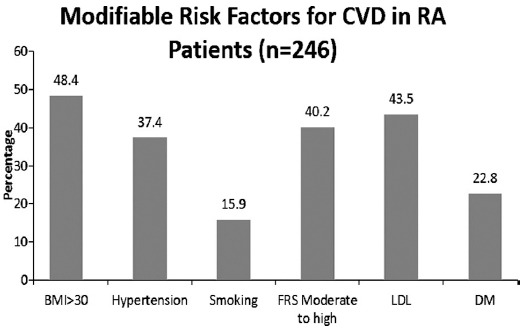
Prevalence of modifiable risk factors.

Most frequent risk factor found in this study was BMI>30 in 48.4% (n=119), High LDL 43.5% (n=107), moderate to high FRS score 40.2% (n=99), Hypertension 37.4% (n=92), Diabetes Mellitus was present in 22.8% (n=56), while smoking was least frequent risk factors with frequency of 15.9% (n=39). (Fig. 1)

**Fig. 2:**
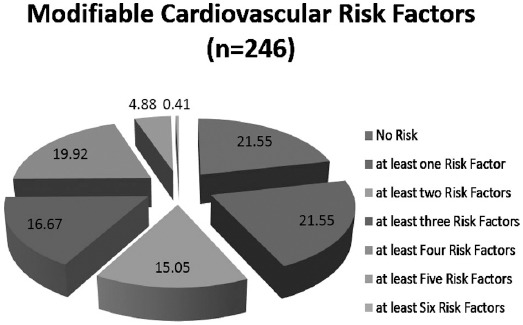
Distribution of risk factors in rheumatoid arthritis.

At least single cardiovascular risk factors was present in 78.48% (n=192) and absent in 21.55% (n=54), while (21.55%) were having at least one risk factors, (15.05%) had two risk factors, (16.67%) had three risk factors, (19.92%) were having four factors, and (4.8%) had five risk factors.(Fig. 2)

Regression analysis was applied to see the effects of disease duration on these risk factors, it was seen that Rheumatoid Arthritis patients having disease duration of seven or more than seven years had more chances to develop modifiable cardiovascular risk factors. Older patients of Rheumatoid Arthritis with disease duration of more than seven years were four times more likely to have High Framingham risk score, moderate to high LDL and diabetes mellitus with significantly high Odds ratio (p-value <0.05) Table-III. Use of Non Methotrexate Disease Modifying anti rheumatoid drugs was also associated with 4 times more risk to develop these modifiable cardiovascular risk factors as well.

**Table-III:** Odds ratio and 95% confidence interval in RA patients for Modifiable and other Risk factors.

*Parameters*		*Odds Ratio*	*95% Confidence Interval*
Age	(Years)	1.13*	(1.08 – 1.18)
Gender	Female	Reference	
	Male	1.65	(0.90 – 3.01)
DMARDS	No DMARDS	Reference	
	MTX	2.50	(0.55 – 11.30)
	SSZ,HQ	1.50	(0.36 – 6.18)
	MTX Combination	1.16	(0.31 – 4.31)
	Non MTX	3.81	(0.90 – 16.19)
LDL	Low	Reference	
	Moderate to High	1.55	(0.93 – 2.58)
BMI	<= 30	Reference	
	>30	1.34	(0.81 – 2.20)
Hypertension	No	Reference	
	Yes	3.84*	(2.01 – 6.03)
Smoking	No	Reference	
	Yes	1.48	(0.73 – 2.96)
FRS	Normal	Reference	
	Moderate to High	3.37*	(2.17 – 6.43)
Diabetes Mellitus	No	Reference	
	Yes	1.85*	(1.0 – 3.41)

* odds considered significant with p<0.05

Age, sex, history of homeopathic medications, High BMI> 30, was also associated with 1-3% more chance to develop modifiable cardiovascular risk factor.

## DISCUSSION

In patients of rheumatoid arthritis the major cause of mortality is cardiovascular diseases not the RA itself, and the main cause of increased risk of CVD is atherosclerosis. Rheumatoid arthritis increases the cardiovascular mortality by up to 50% compared with general population, interestingly the pattern of cardiovascular diseases is different too, as most of patient suffer silent myocardial infarction and ischemic heart disease and experience sudden death. Traditional cardiovascular risk factors hypertension, Type-2 diabetes, smoking, hyperchloestremia, obesity and physical inactivity contributes to the excessive CVD risk to some extent, it is the high systemic inflammatory burden associated with RA appears to be decisive factor.^6^

Despite of recent improvements in treatment the gap between RA patients and general populations in terms of mortality secondary to cardiovascular complications is not decreasing so it is important to control baseline cardiovascular risk.^7^ Sharmayne et al in their cross sectional control study found that patients with RA had higher BMI (28.05) than controls (26.25).^8^

In our study we also found higher mean BMI (29.03) in both sexes, while males were more in over weight and obese groups. In this study the mean Systolic blood pressure was (126-131 mmHg) and means diastolic blood pressure was (82-85mmHg), study by Shermayne et al the mean Systolic Blood pressure was (148.08mmHg) and mean diastolic Blood pressure was (82.2mmHg).

In this study the elevated BMI was (48.4%), Hypertension (37.4%), smoking (15.9%), FRS score of moderate to high (40.2%) and Diabetes mellitus (22.8%) while Chung et al study the prevalence of elevated BMI was (35%), DM (7%), Hypertension (57%), and smoking was (12%).^3^ Jean-Frederic Boyer et al in their meta-analysis noted the increased prevalence of Diabetes, smoking and lower HDL cholesterol levels in RA patients.^9^ VP Van Halem in cross sectional study result showed that when compared with non-diabetic controls the Odds Ratio for CVD was 3 times higher in RA group and 2.5 times higher in DM2 group.^10^ Adding to classical cardiovascular risk factors chronic inflammation and disease distribution contribute decisively to the excess of cardiovascular mortality in RA so EULAR recommends annual cardiac risk assessment in such patients.^11^ Higher number of elevated BMI and Diabetes mellitus cases in our study population seems to be because of steroid use through hakeem medications, sedentary life style, disease burden and NSAID’s use.

We found use of non-methotrexate disease modifying anti rheumatoid drugs was also associated with four times more risk to develop these modifiable cardiovascular risk factors.

### Limitations

This study has few limitations first it’s a cross sectional study, no comparative group, but this is first study carried out on rheumatoid arthritis patients in Pakistan. In this study we used Framingham risk stratification model but currently ACC/AHA guidelines recommend pooled Cohort Equation for risk stratification in non-African American and non-Hispanic population but this guideline is silent about the risk stratification model for auto immune diseases.

## CONCLUSION

Due to increased mortality and morbidity related with cardiovascular events in the like Myocardial infarction, angina, congestive heart failure, stroke, transient ischemic attacks, in Rheumatoid arthritis, these patients should be encouraged to avoid sedentary life style, use healthy diet, remain physically active as much possible, minimize illicit drug use, quit smoking, and yearly cardiovascular risk score estimation to be done.
